# Supplementary material for: Visualizing structural transitions of ligand-dependent gating of the TRPM2 channel
Source: Nat Commun. 2019 Aug 20;10:3740. doi: 10.1038/s41467-019-11733-5 (PMC6702222; doi:10.1038/s41467-019-11733-5)
Supplement: Supplementary file 1 — Supplementary Information [file 41467_2019_11733_MOESM1_ESM.docx]

**Supplementary Information**

**Visualizing structural transitions of**

**ligand-dependent gating of the TRPM2 channel**

Y. Yin *et al.*


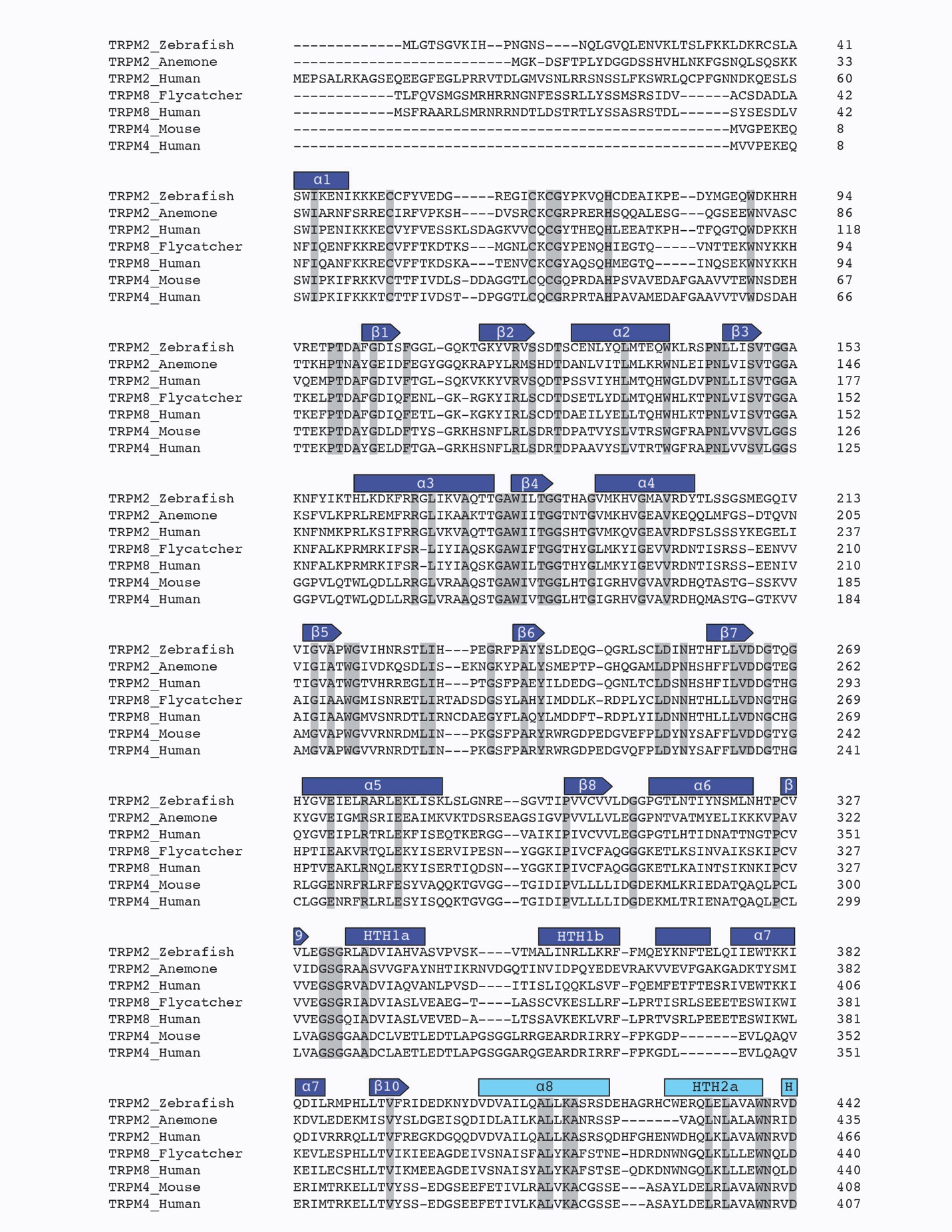


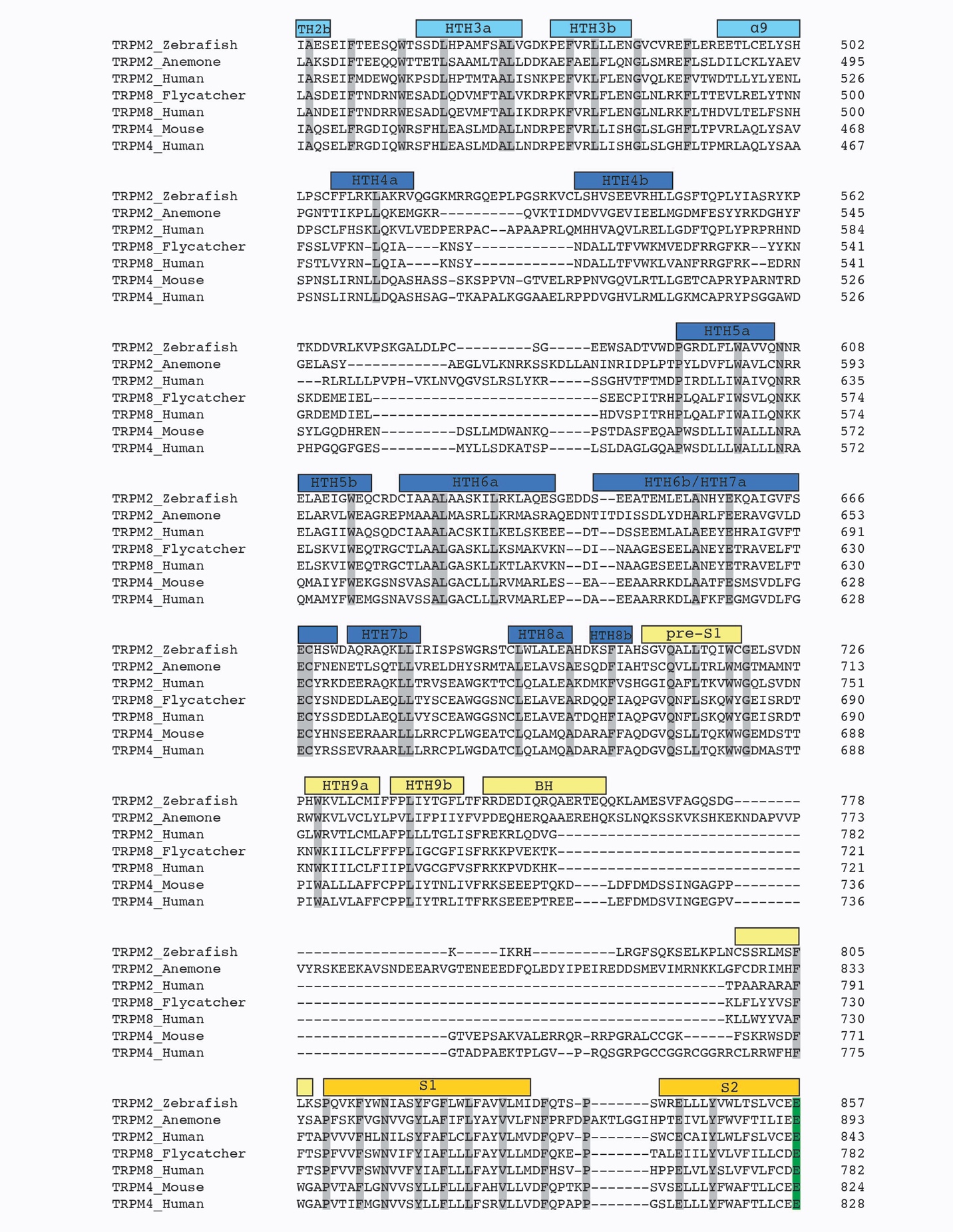


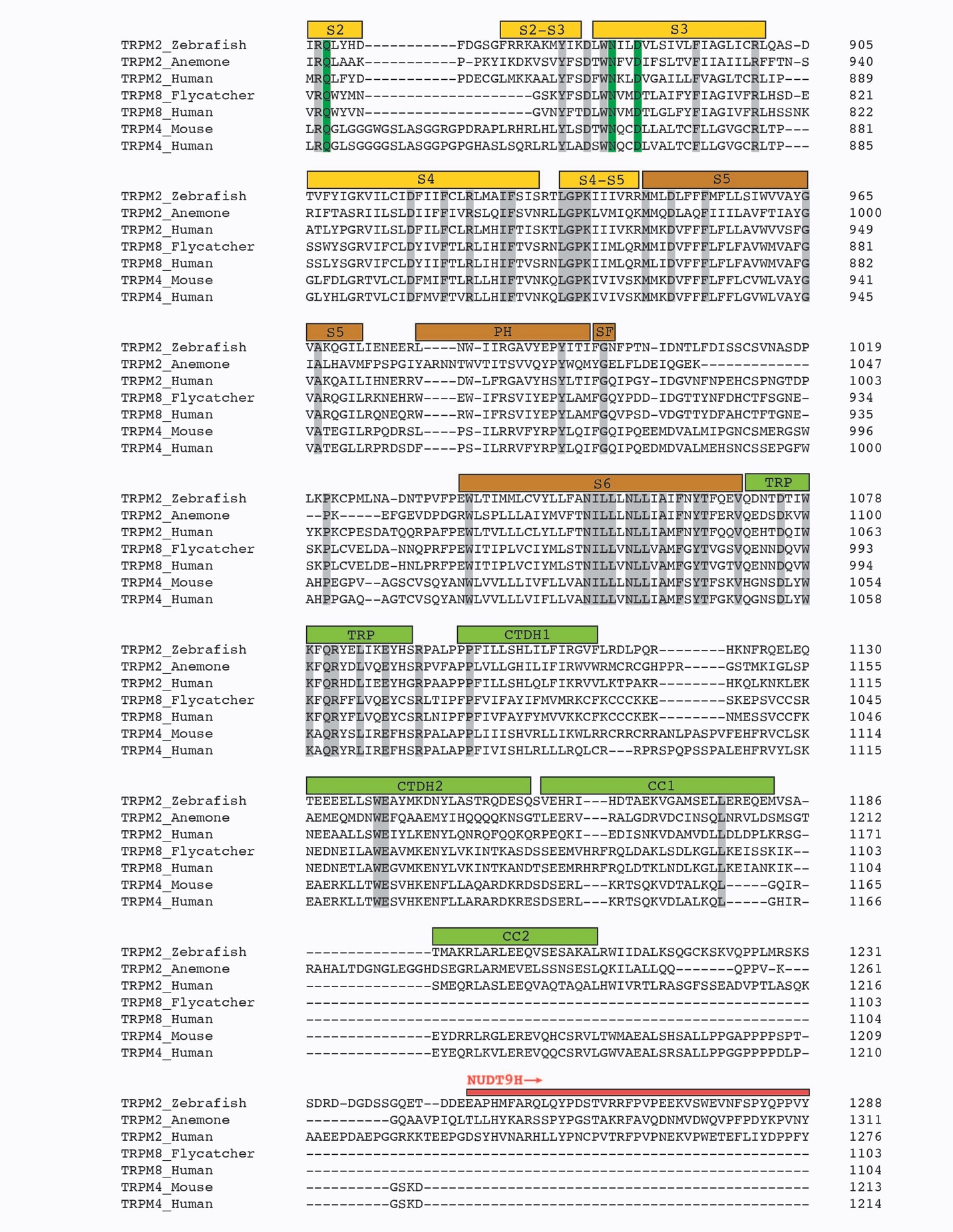


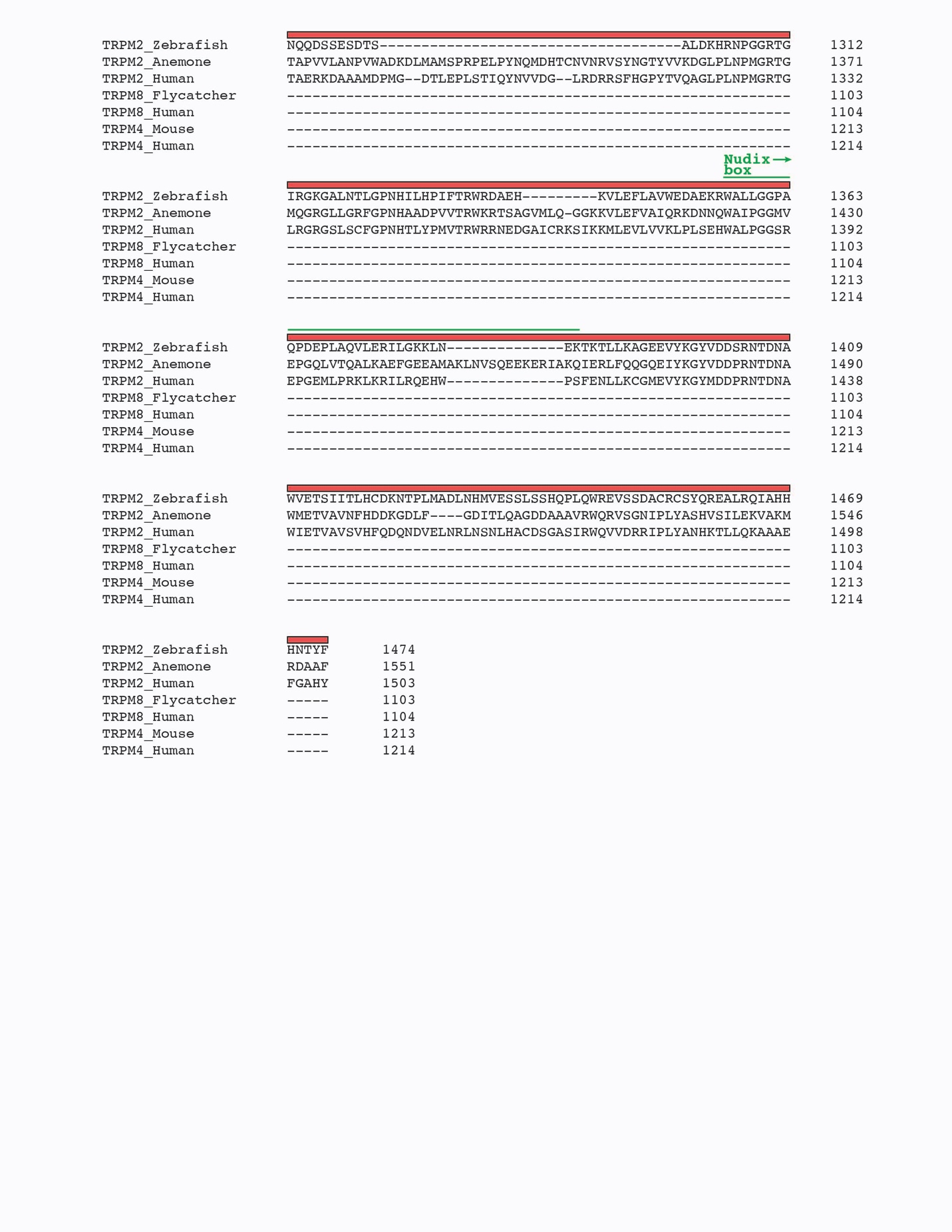


**Supplementary Figure 1. Sequence alignment of TRPM2 with representative TRPM8 and TRPM4 orthologues.**

Secondary structures in the TRPM2_DR_ structure are indicated by rectangles (helices) and arrows (β-strands) and are colored as in Fig. 1d. Residues with absolute conservation are highlighted in gray. Sequences corresponding to the NUDT9H domain in zebrafish and human TRPM2 channels are indicated by red bars. Green line identifies the Nudix box.


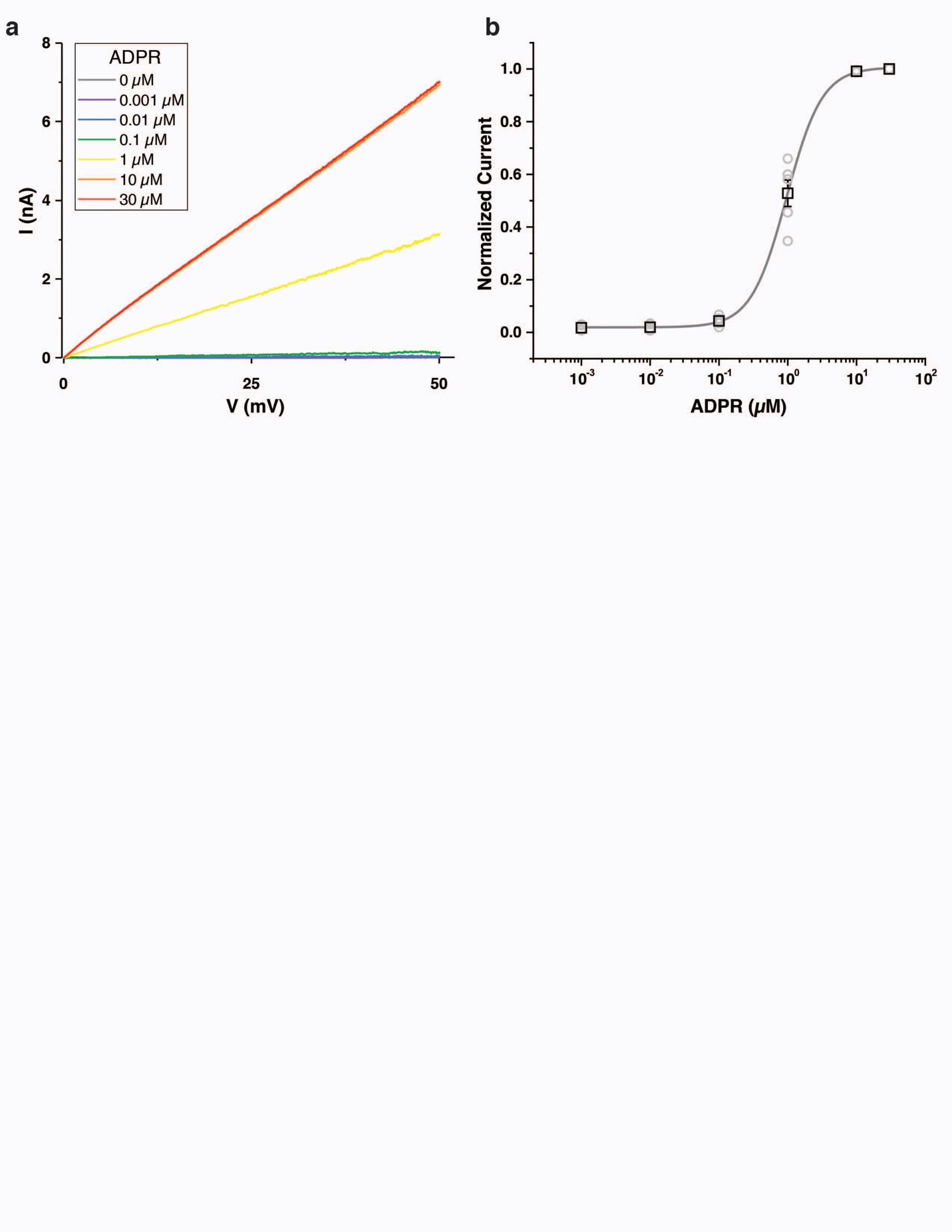


**Supplementary Figure 2. Functional characterization of the TRPM2_DR_ channel.**

**a**, Representative inside-out current traces recorded with repeated voltage ramps (from 0 to +50 mV; 400 ms) from HEK293T cells transfected with TRPM2_DR_ during application of 0 μM (gray), 0.001 μM (purple), 0.01 µM (blue), 0.1 μM (green), 1 µM (yellow), 10 µM (orange), and 30 μM (red) ADPR to the inside of the patch membrane in the presence of 125 μM Ca^2+^.

**b**, ADPR dose-response relationship of TRPM2_DR_ currents measured at +50 mV (V_m_ = -50 mV) generated from five samples (n = 5; biologically independent experiments, gray circles) and average values (black squares and error bars denote means ± SEM) fit with the Hill equation (dark gray curve). The half-maximal ADPR concentration (EC_50_) was 0.96 ± 0.01 µM with a Hill coefficient (n_H_) of 1.7 ± 0.1. The source data underlying Supplementary Fig. 2b are provided as a Source Data file.


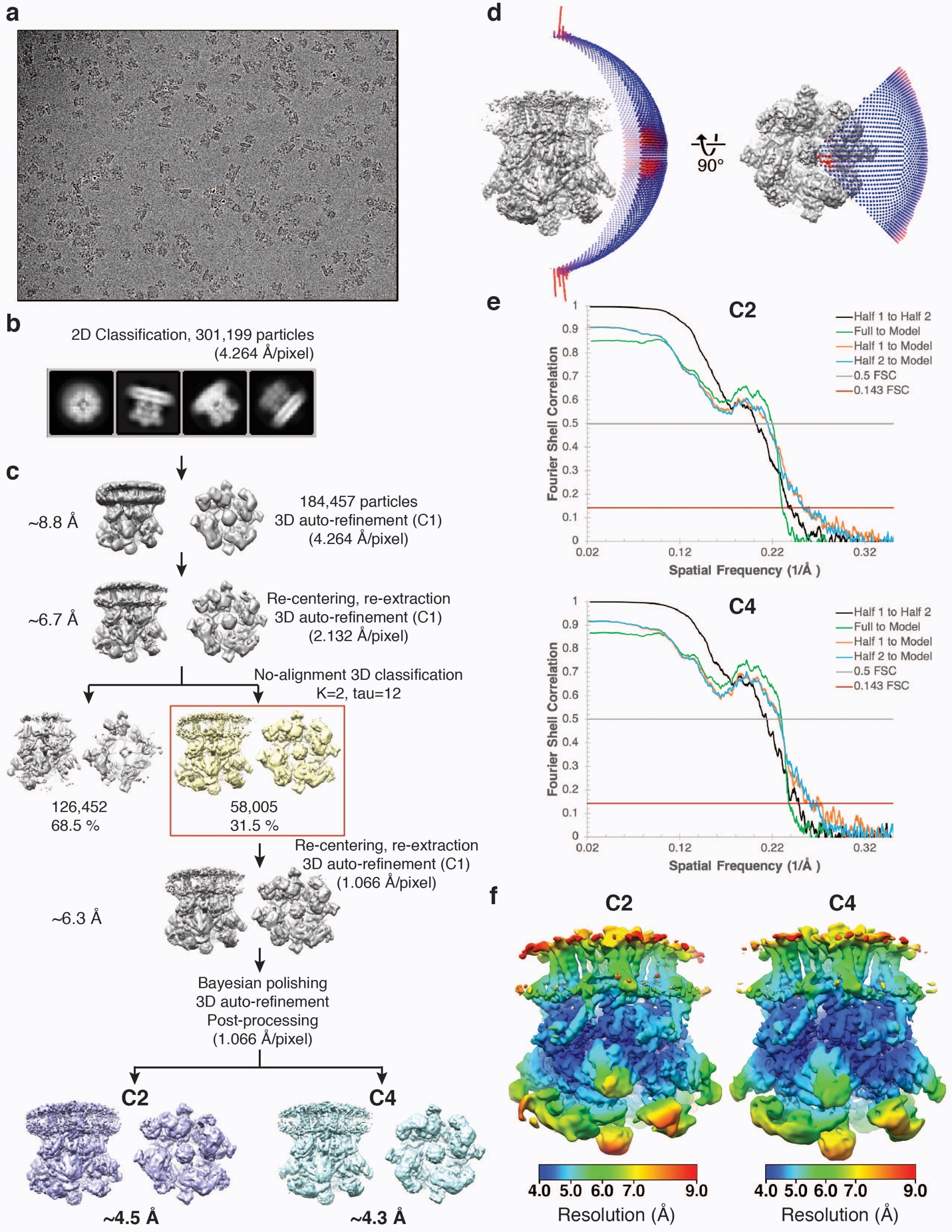


**Supplementary Figure 3. Cryo-EM data processing of TRPM2_DR_Apo_ structure.**

a, Representative micrograph of TRPM2_DR_Apo_ sample in vitreous ice. 3776 movies were collected.

**b**,**c**, Particles comprising the good 2D classes (**b**) were 3D auto-refined to a reconstruction of ~8.8 Å with C1 symmetry. Refined 184,457 particles were re-extracted, re-centered, Fourier binned 2 x 2, 3D auto-refined with C1 symmetry and subjected to 3D classification without alignment. For individual class, the number of particles and the percentage relative the total number of particles input to the classification are listed. 58,005 particles comprising the 3D class, in which the TMD and CD were better resolved, were re-centered, re-extracted, and unbinned, and were subjected to 3D auto-refinement with C1 symmetry, followed by Bayesian polishing. The shiny particles were input to a final round of 3D auto-refinement with C2 and C4 symmetry in parallel, yielding a final reconstruction of ~4.5 Å and ~4.3 Å, respectively (**c**).

**d**, Euler distribution plot for the final 3D reconstruction of the TRPM2_DR_Apo-C4_ structure.

**e**, FSC curves calculated between the half maps (black line), molecular model and the full map (green line), and between the model and each half-map (orange and blue lines), for the TRPM2_DR_Apo-pseudo C4_ (top) and TRPM2_DR_Apo-C4_ (bottom) structures, respectively.

**f**, Local resolution estimation of the final 3D reconstruction for the TRPM2_DR_Apo-pseudo C4_ (left) and TRPM2_DR_Apo-C4_ (right) structures, respectively. RELION-3.0 was used for calculation.


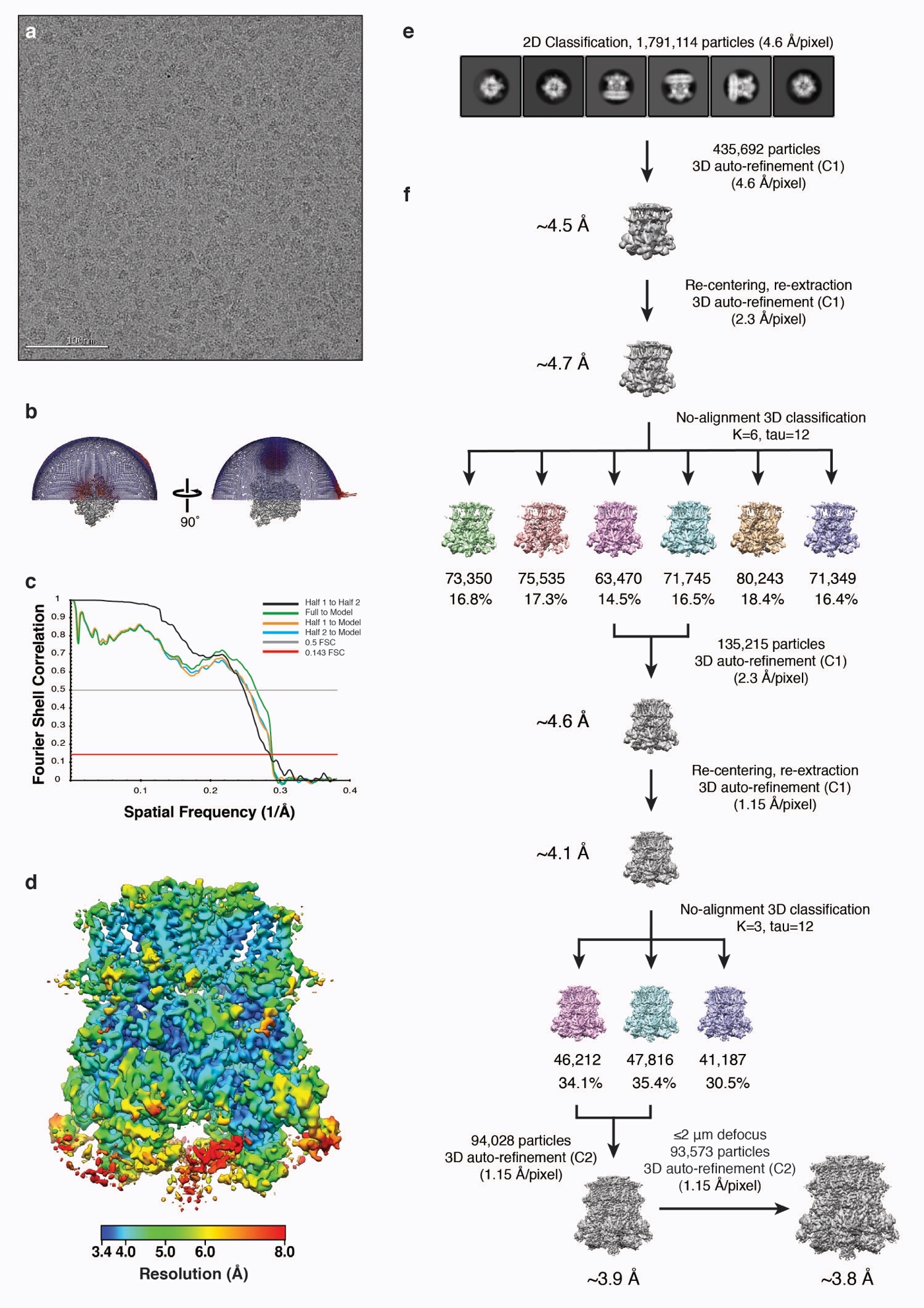


**Supplementary Figure 4. Cryo-EM data processing of TRPM2_DR_Ca2+_ structure.**

**a**, Representative micrograph of TRPM2_DR_Ca2+_ in vitreous ice. 3,039 movies of TRPM2 were collected over continuous carbon.

**b**, Euler distribution plot for the final reconstruction.

**c**, FSC curves calculated between the half maps (black line), atomic model and the final map (green line), and between the model and each half-map (orange and blue lines).

**d**, Local resolution estimates of the final reconstructions calculated using BSOFT ^1^.

**e**,**f**, 1,791,114 particles were extracted from aligned micrographs, Fourier binned 4 x 4, and subjected to reference-free 2D classification using RELION. Representative 2D class averages are shown (**e**). Particles comprising the “best” class averages were 3D auto-refined without symmetry to yield a ~4.5 Å resolution reconstruction. Refined particle coordinates were used for re-centering and extraction of particles. Particles were Fourier binned by 2 x 2, followed by 3D auto-refinement and no-alignment 3D classification. For each class, the number of contributing particles and percentage relative to total particles input to classification are listed below, respectively. 135,215 particles corresponding to the best-resolved classes were combined and 3D auto-refined to yield a ~4.6 Å resolution reconstruction. Refined particle coordinates were re-centered and extracted unbinned, auto-refined, and subjected to no- alignment classification to obtain a subset of 94,028 particles. Particles collected at greater than 2 μm defocus were removed to obtain a final particle stack of 93,573 particles, which was auto- refined with C2 symmetry enforced to yield a final reconstruction at ~3.8 Å resolution (**f**).

**
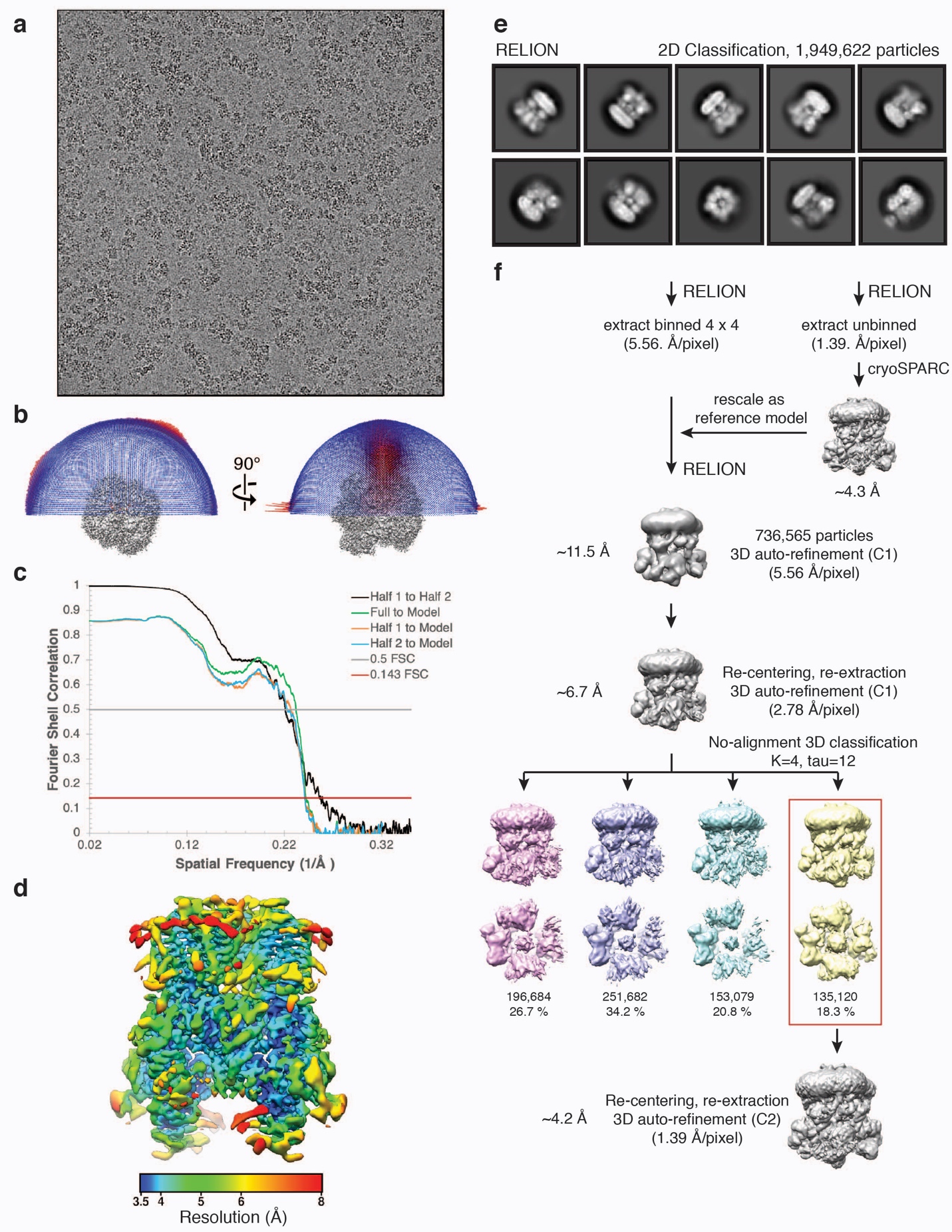
**

**Supplementary Figure 5. Cryo-EM data processing of TRPM2_DR_ADPR/Ca2+_ structure.**

a, Representative micrograph of TRPM2_DR_ADPR/Ca2+_ sample in vitreous ice. 2496 movies were collected.

**b**, Euler distribution plot for the final reconstruction.

**c**, FSC curves calculated between the half maps (black line), molecular model and the full map (green line), and between the model and each half-map (orange and blue lines).

**d**, Local resolution estimation of the final reconstruction calculated using BSOFT ^1^.

**e**, Particles comprising the good 2D classes were 3D auto-refined to a reconstruction of ~11.5 Å with C1 symmetry using the 3D reconstruction generated by cryoSPARC as the reference model. Refined 736,565 particles were re-extracted, re-centered, Fourier binned 2 x 2, 3D auto-refined with C1 symmetry and subjected to no-alignment 3D classification. For individual class, the number of particles and the percentage relative the total number of particles input to the classification are listed. 135,120 particles comprising the 3D class, in which the cytoplasmic domain (CD) is most well-resolved, were re-centered, re-extracted, and unbinned, and were subjected to 3D auto-refinement with C2 symmetry, yielding a final reconstruction of ~4.2 Å.


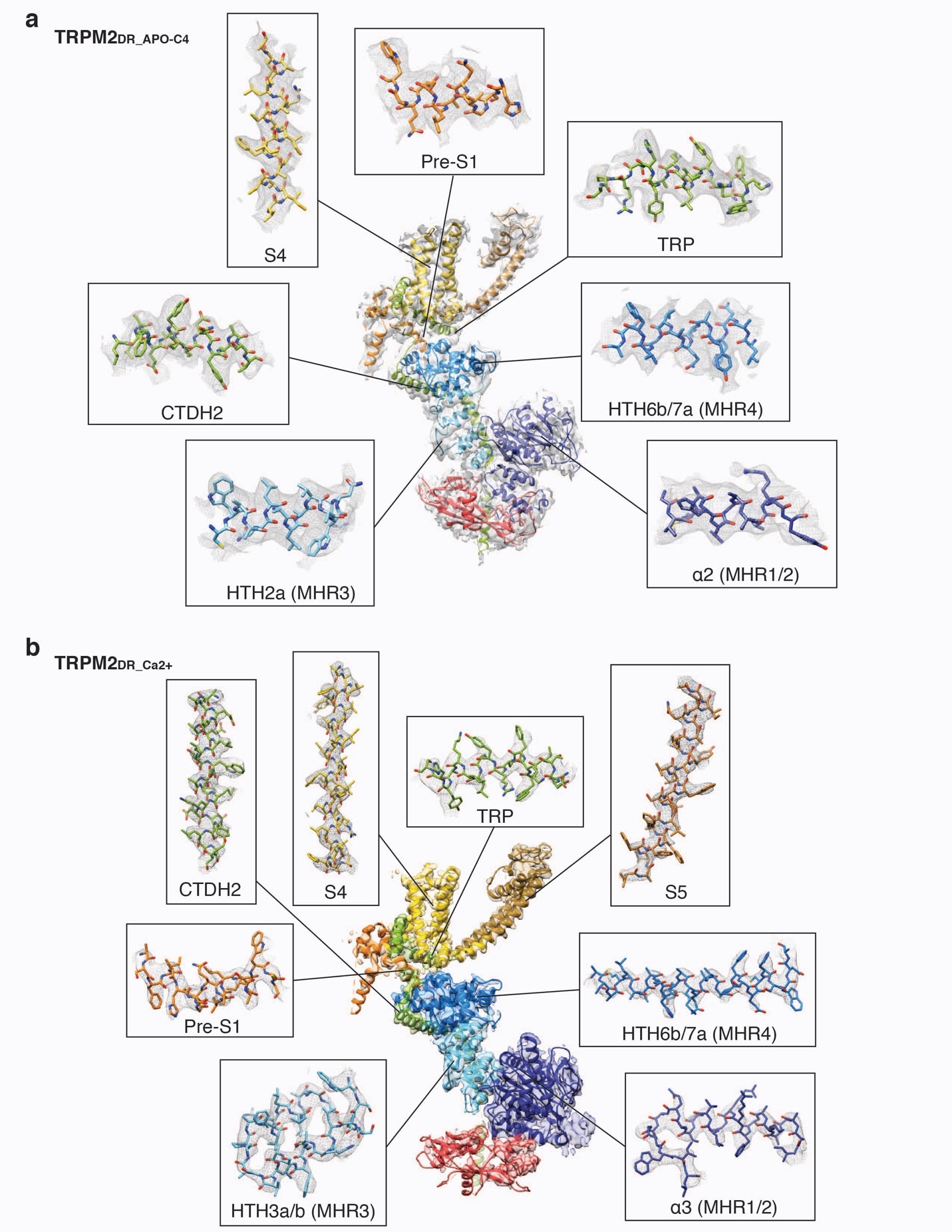

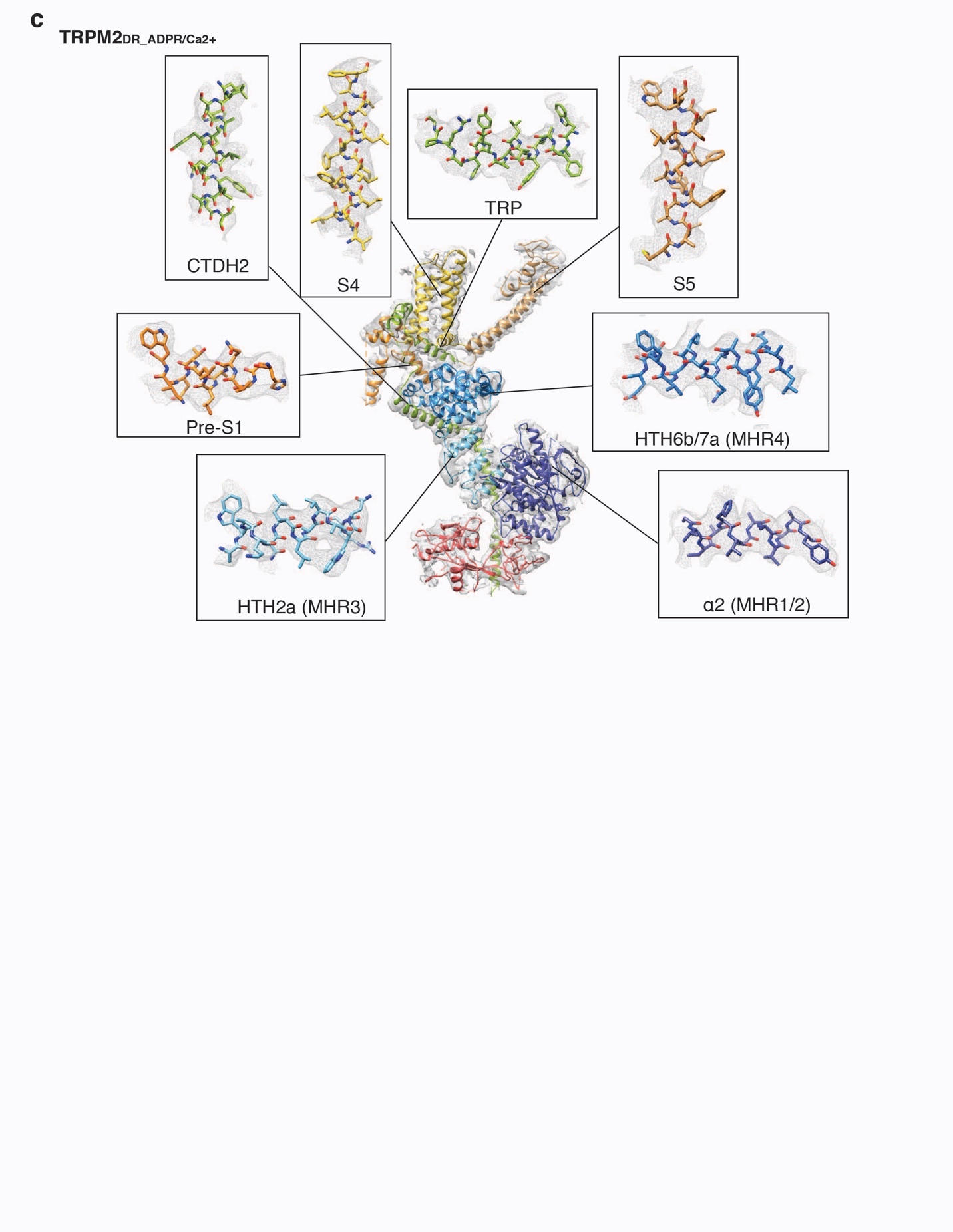


**Supplementary Figure 6. Quality of electron density of key structural elements.**

**a-c**, The structural elements in the TRPM2_DR_Apo-C4_ (**a**), TRPM2_DR_Ca2+_ (**b**), and TRPM2_DR_ADPR/Ca2+_ (**c**) structures are labeled and colored according to Fig. 1d and shown as sticks. The electron density is shown as gray mesh, zoned ~2.5 Å, ~2 Å, and ~2.5 Å around atoms in (**a**), (**b**), and (**c**), respectively.


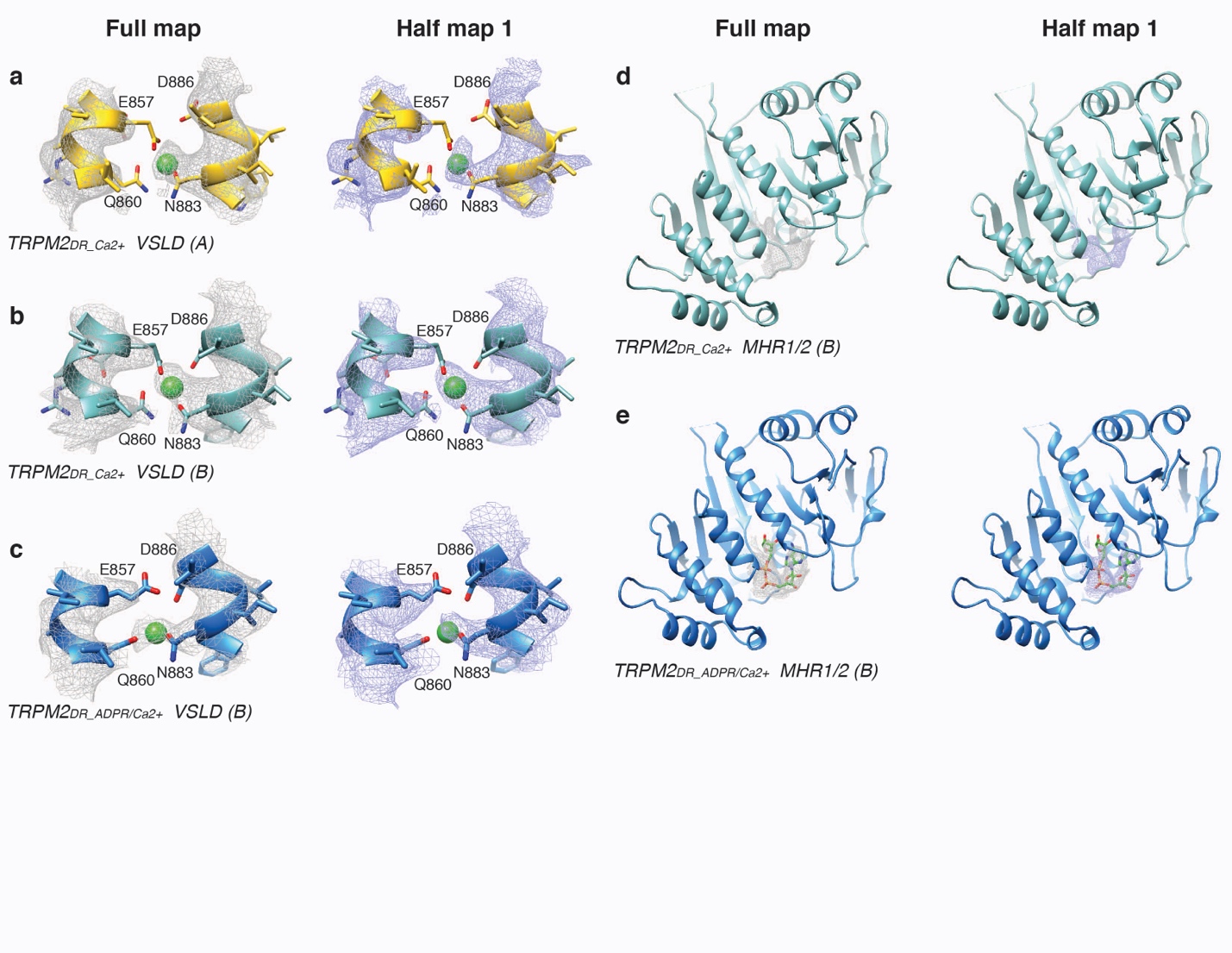


**Supplementary Figure 7. Cryo-EM densities of Ca^2+^ and ADPR in the TRPM2_DR_ structures.**

**a**, Cryo-EM density of the Ca^2+^ ion located in the VSLD cavity of the protomer A in the TRPM2_DR_Ca2+_ structure, in the full map (left, gray mesh) and half map 1 (right, purple mesh) zoned ~2.5 Å around atoms at 0.045 and 0.025 thresholding.

**b**, Cryo-EM density of the Ca^2+^ ion located in the VSLD cavity of the protomer B in the TRPM2_DR_Ca2+_ structure, in the full map (left, gray mesh) and half map 1 (right, purple mesh) zoned ~2.5 Å around atoms at 0.045 and 0.025 thresholding.

**c**, Cryo-EM density of the Ca^2+^ ion located in the VSLD cavity of the protomer B in the TRPM2_DR_ADPR/Ca2+_ structure, in the full map (left, gray mesh) and half map 1 (right, purple mesh) zoned ~2.5 Å around atoms at 0.05 thresholding.

**d,** Putative EM density resembling ADPR located in the MHR1/2 domain of the protomer B in the TRPM2_DR_Ca2+_ structure, in the full map (left, gray mesh) and half map 1 (right, purple mesh) zoned ~2.5 Å around atoms at 0.05 and 0.035 thresholding.

**e**, Cryo-EM density of the ADPR located in the MHR1/2 domain of the protomer B in the TRPM2_DR_ADPR/Ca2+_ structure, in the full map (left, gray mesh) and half map 1 (right, purple mesh) zoned ~2.5 Å around atoms at 0.045 thresholding.


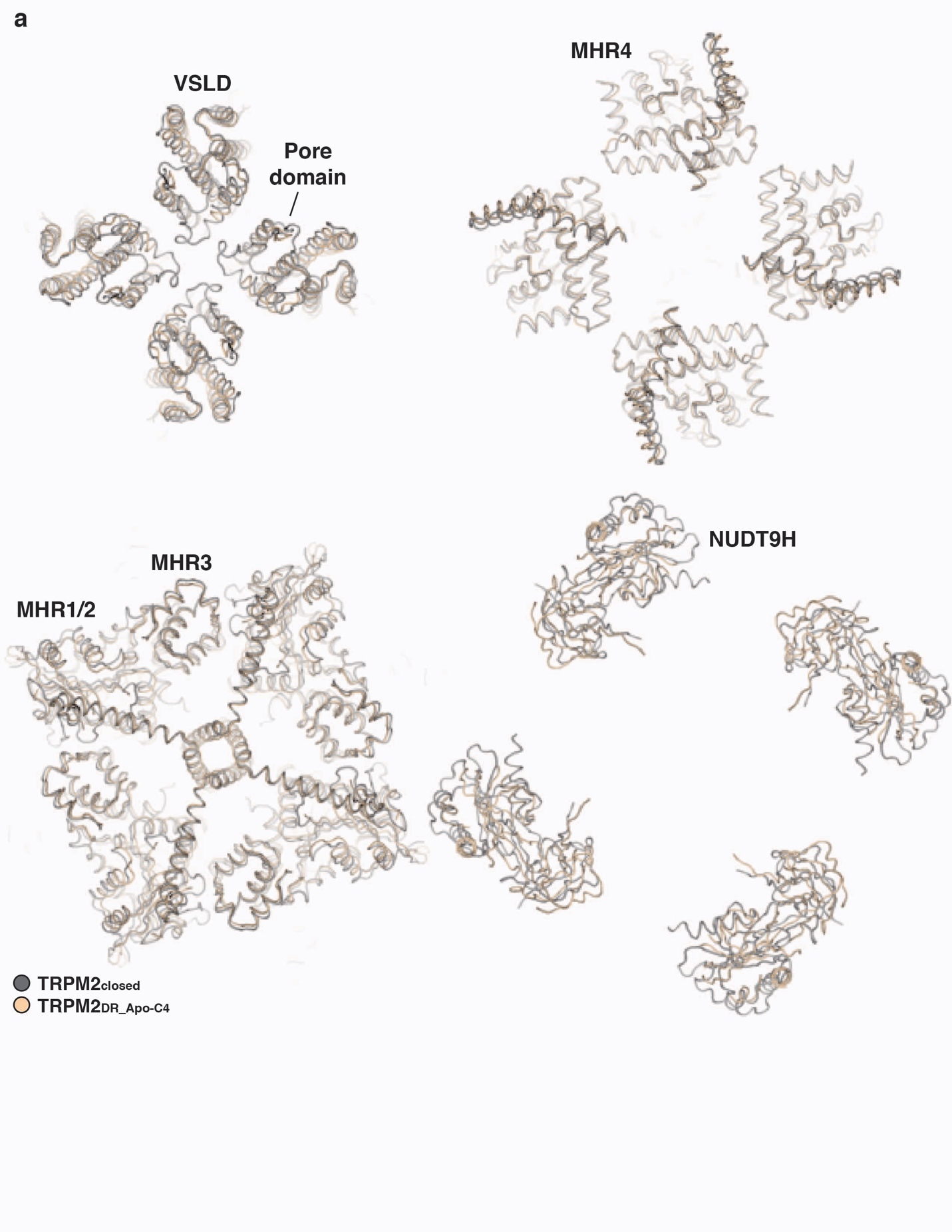

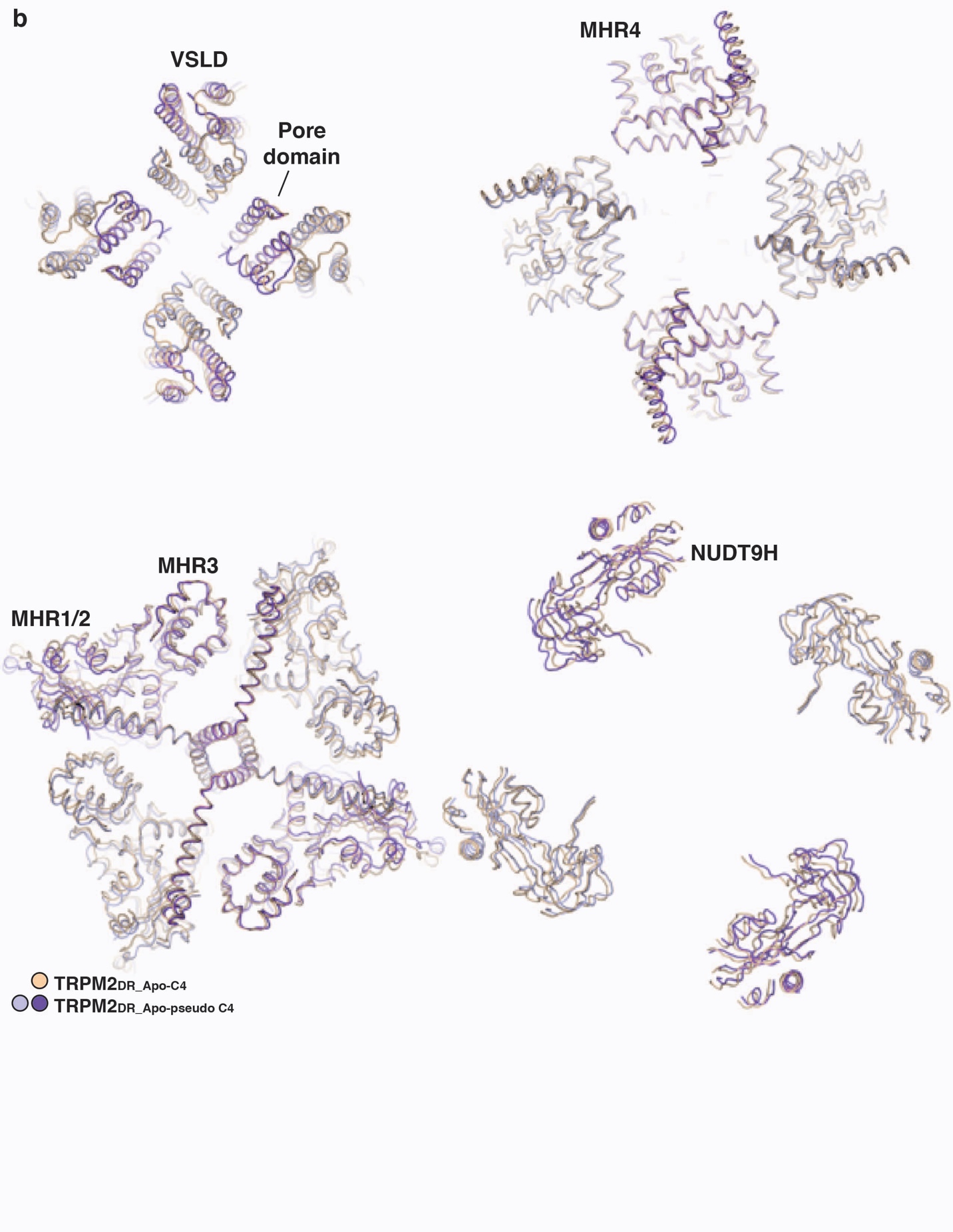


**Supplementary Figure 8. Comparison of symmetry for the TRPM2_DR_Apo_ structure.**

**a**, Top views showing comparison of the TRPM2_DR_Apo-C4_ (wheat) and TRPM2_closed_ (silver, PDB 6DRK) tetramers at different layers of the channel.

**b**, Top views showing comparison of the TRPM2_DR_Apo-C4_ (wheat) and TRPM2_DR_Apo-pseudo C4_ (purple) tetramers at different layers of the channel.

**
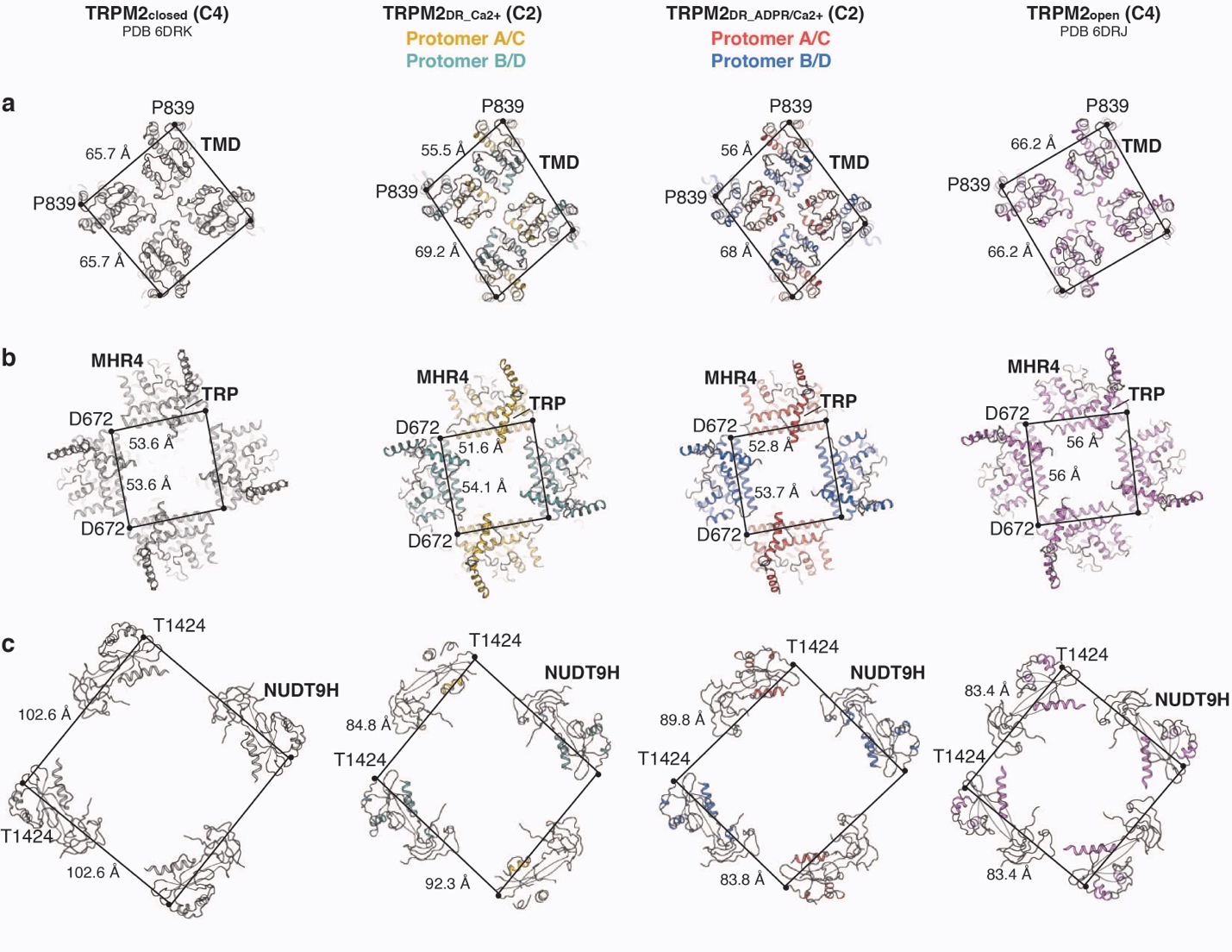
**

**Supplementary Figure 9. Comparison of the channel symmetry in TRPM2_DR_ structures.**

**a**-**c**, Extracellular views of the TMDs (**a**), sliced between the TMDs and the CDs (**b**), and between the middle and bottom layer of the CDs (**c**) in the TRPM2_closed_, TRPM2_DR_Ca2+_, TRPM2_DR_ADPR/Ca2+_, and TRPM2_open_ structures, comparing the two-fold symmetry in the TRPM2_DR_Ca2+_ and TRPM2_DR_ADPR/Ca2+_ structures with the four-fold symmetry in the TRPM2_closed_ and TRPM2_open_ structures.


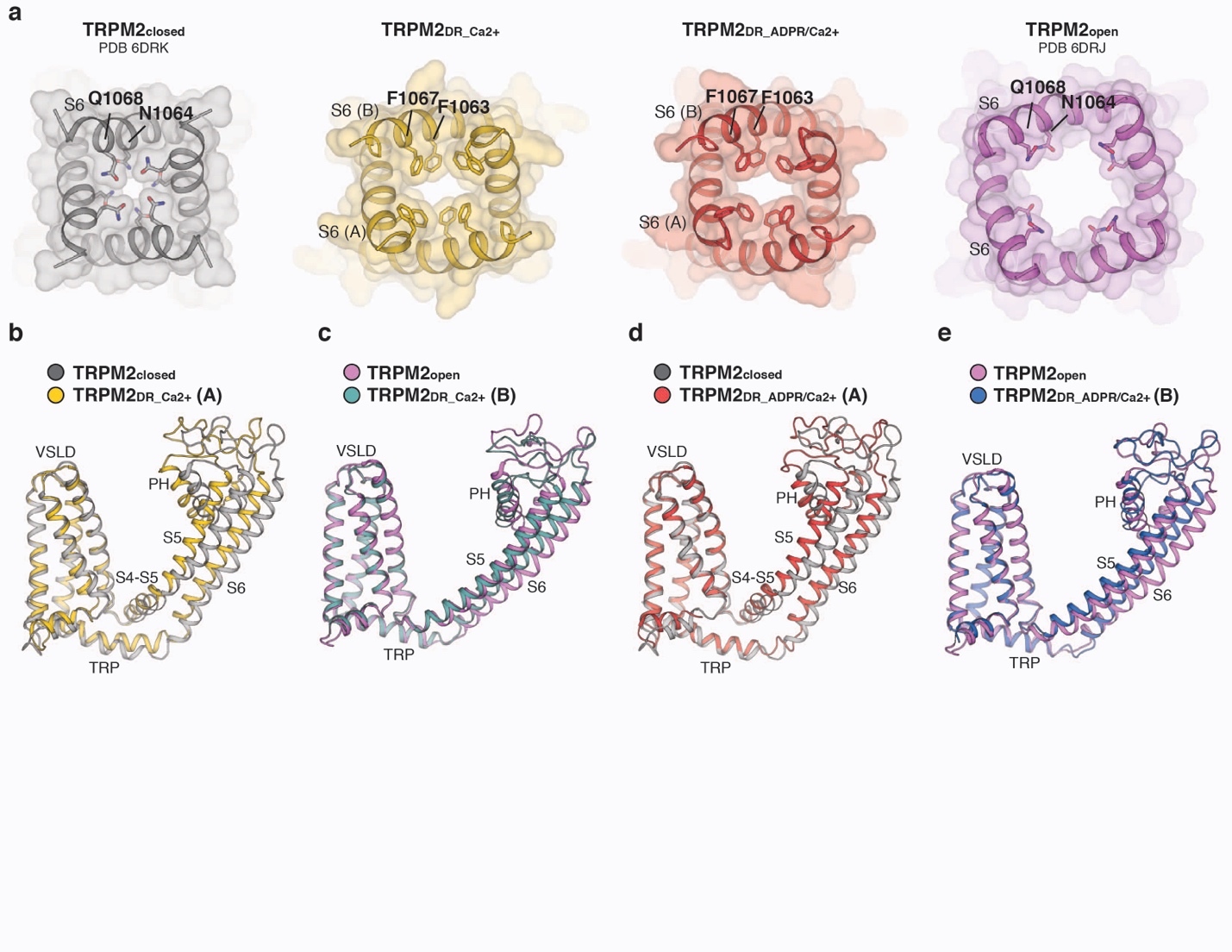


**Supplementary Figure 10. Comparison of the transmembrane channel domain (TMD) in TRPM2_DR_ structures.**

**a**, Cartoon and surface representations of the S6 gate viewed from the intracellular side. In the TRPM2_closed_ (silver) and TRPM2_open_ (violet) structures, residues N1064 and Q1068 form the narrowest restriction points, while residues F1063 and F1067 on S6 form the intracellular gate in the TRPM2_DR_Ca2+_ (yellow) and TRPM2_DR_ADPR/Ca2+_ (red) structures.

**b**, the TMD of protomer A in the TRPM2_DR_Ca2+_ structure (yellow) resembles that of the TRPM2_closed_ structure (silver).

**c**, the TMD of protomer B in the TRPM2_DR_Ca2+_ structure (teal) resembles that of the TRPM2_open_ structure (violet).

**d**, the TMD of protomer A in the TRPM2_DR_ADPR/Ca2+_ structure (red) resembles that of the TRPM2_closed_ structure (silver).

**e**, the TMD of protomer B in the TRPM2_DR_ADPR/Ca2+_ structure (blue) resembles that of the TRPM2_open_ structure (violet).


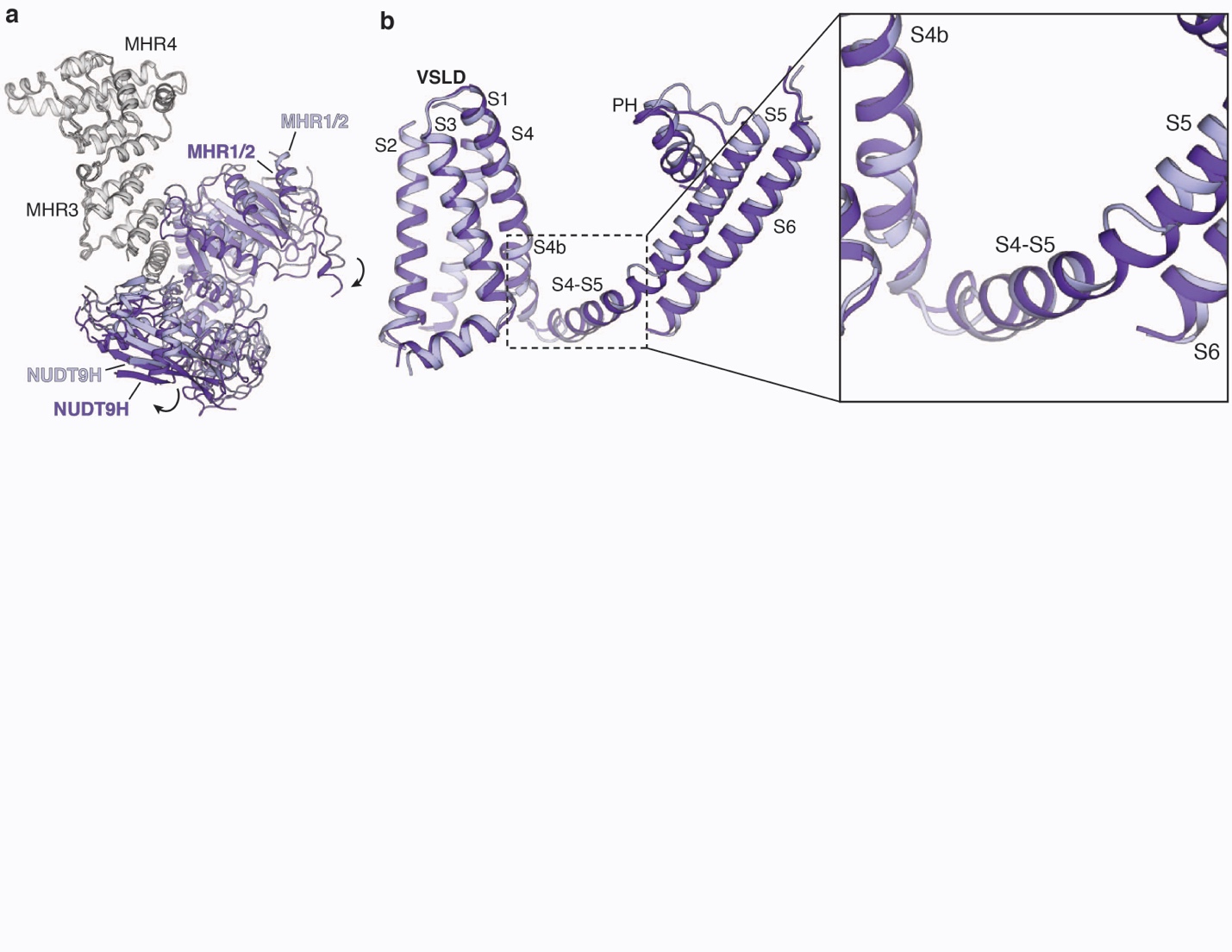


**Supplementary Figure 11. Structural deviations between protomers A and B of the TRPM2_DR_Apo-pseudo C4_ structure.**

**a**, Protomers A (light purple) and B (purple) aligned at MHR3 and MHR4 domains (gray).

**b**, Protomers A (light purple) and B (purple) aligned at VSLD. Close-up view showing slight structural divergence at S4b and S4-S5 linker regions, giving rise to the deviation from C4 symmetry in the TMD.


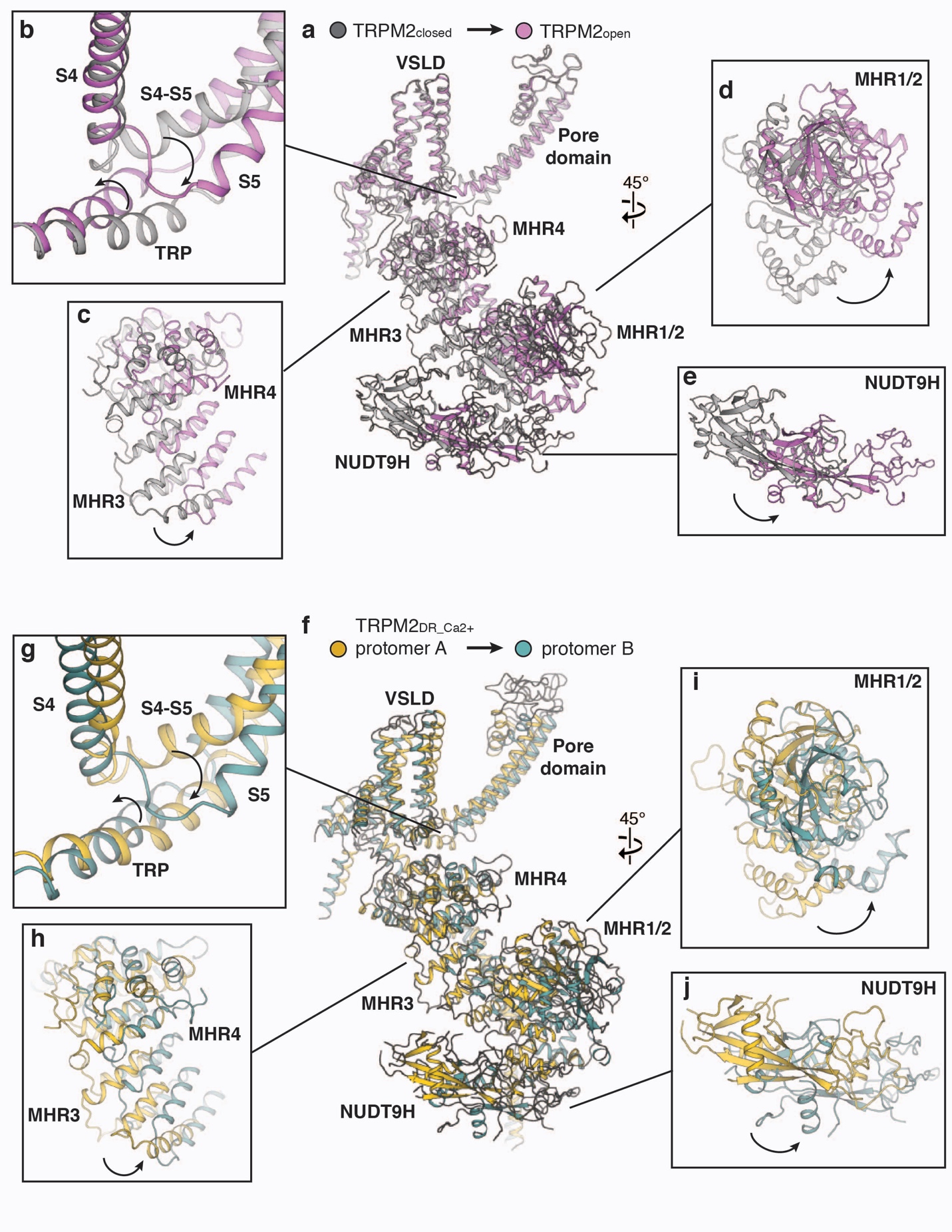


**Supplementary Figure 12. Conformational rearrangements during TRPM2 channel gating.**

**a**, Viewed from the membrane plane, cartoon representations of TRPM2_closed_ and TRPM2_open_ protomers aligned at the TMDs, showing how conformational changes in the CD could be propagated to TMD during channel activation. Protomers of TRPM2_closed_ and TRPM2_open_ are colored in silver and violet, respectively.

**b**-**e**, Close-up views of the structural rearrangements of TRP domain and S4-S5 linker (**b**), MHR3-4 (**c**), MHR1/2 (**d**), and the NUDT9H domain (**e**) depicted in (**a**).

**f**, Viewed from the membrane plane, cartoon representations of protomers A and B of TRPM2_DR_Ca2+_ structure aligned at the TMDs, showing conformational changes propagated from CD to TMD resemble those depicted in (**a**). Protomers A and B are colored in gold and teal, respectively.

**g**-**j**, Close-up views of the structural rearrangements of TRP domain and S4-S5 linker (**g**), MHR3-4 (**h**), MHR1/2 (**i**), and the NUDT9H domain (**j**) depicted in (**f**).


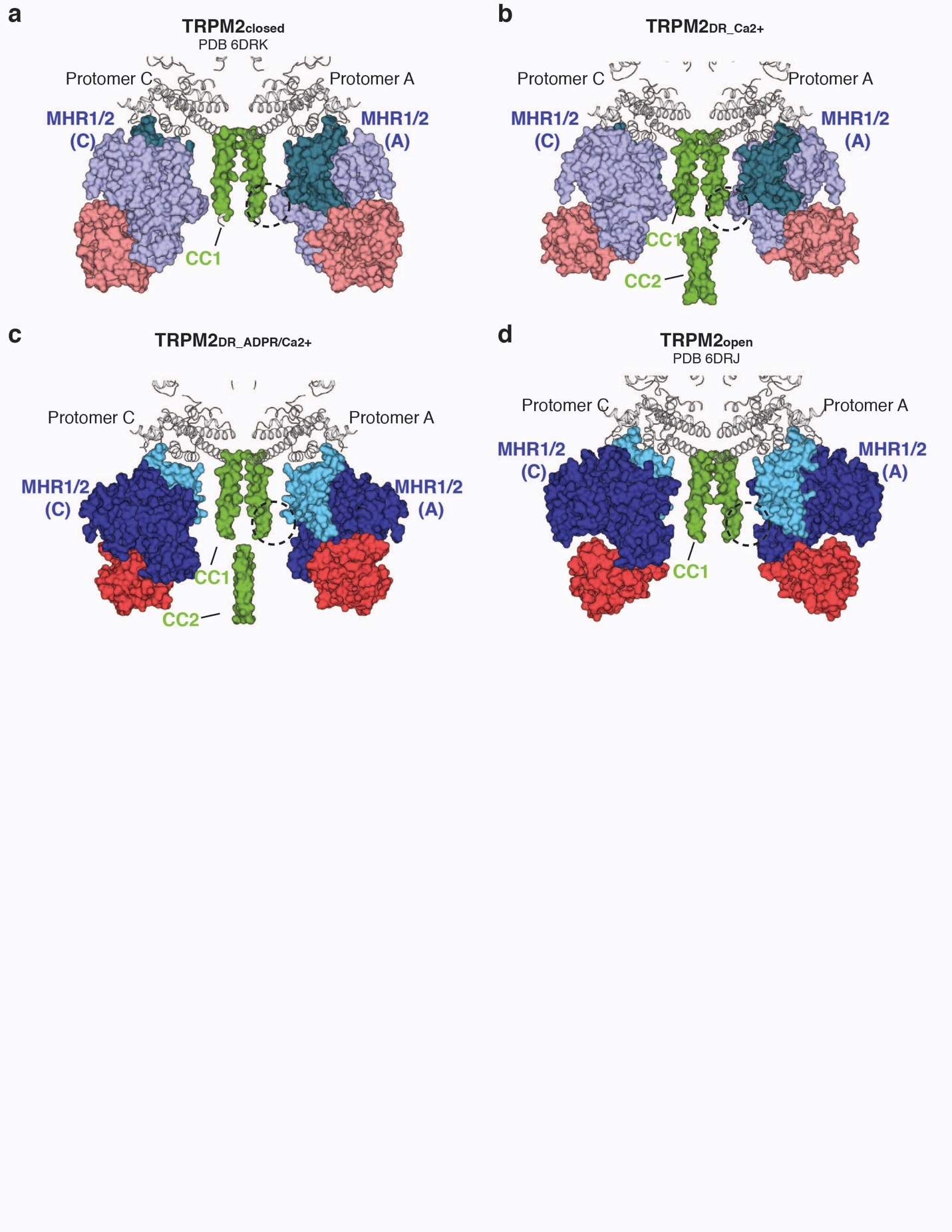


**Supplementary Figure 13. Comparison of the interfacial contact between CC1 and MHR1/2 domain in different TRPM2_DR_ structures.**

**a**-**d**, Cross-section views of opposing protomers A and C in the published TRPM2_closed_ structure (**a**, PDB 6DRK), in TRPM2_DR_Ca2+_ (**b**) and TRPM2_DR_ADPR/Ca2+_ (**c**) structures from the current study, and in the published TRPM2_open_ structure (**d**, PDB 6DRJ), showing the detachment of MHR1/2 domain from CC1 in the TRPM2_DR_ADPR/Ca2+_ (**c**) and TRPM2_open_ (**d**) structures, in contrast with the association between the two domains in the TRPM2_closed_ (**a**) and TRPM2_DR_Ca2+_. (**b**) structures.

| Amino acid sequence |
| --- |
| MALGTSGVKIHPNGNSNQLGVQLENVKLTSLFKKLDKRCSLASWIKENIKKKECCFYVEDGREGICKCGYPKVQHCDEAIKPEDYMGEQWDKHRHVRETPTDAFGDISFGGLGQKTGKYVRVSSDTSCENLYQLMTEQWKLRSPNLLISVTGGAKNFYIKTHLKDKFRRGLIKVAQTTGAWILTGGTHAGVMKHVGMAVRDYTLSSGSMEGQIVVIGVAPWGVIHNRSTLIHPEGRFPAYYSLDEQGQGRLSCLDINHTHFLLVDDGTQGHYGVEIELRARLEKLISKLSLGNRESGVTIPVVCVVLDGGPGTLNTIYNSMLNHTPCVVLEGSGRLADVIAHVASVPVSKVTMALINRLLKRFFMQEYKNFTELQIIEWTKKIQDILRMPHLLTVFRIDEDKNYDVDVAILQALLKASRSDEHAGRHCWERQLELAVAWNRVDIAESEIFTEESQWTSSDLHPAMFSALVGDKPEFVRLLLENGVCVREFLEREETLCELYSHLPSCFFLRKLAKRVQGGKMRRGQEPLPGSRKVCLSHVSEEVRHLLGSFTQPLYIASRYKPTKDDVRLKVPSKGALDLPCSGEEWSADTVWDPGRDLFLWAVVQNNRELAEIGWEQCRDCIAAALAASKILRKLAQESGEDDSEEATEMLELANHYEKQAIGVFSECHSWDAQRAQKLLIRISPSWGRSTCLWLALEAHDKSFIAHSGVQALLTQIWCGELSVDNPHWKVLLCMIFFPLIYTGFLTFRRDEDIQRQAERTEQQKLAMESVFAGQSDGKIKRHLRGFSQKSELKPLNCSSRLMSFLKSPQVKFYWNIASYFGFLWLFAVVLMIDFQTSPSWRELLLYVWLTSLVCEEIRQLYHDFDGSGFRRKAKMYIKDLWNILDVLSIVLFIAGLICRLQASDTVFYIGKVILCIDFIIFCLRLMAIFSISRTLGPKIIIVRRMMLDLFFFMFLLSIWVVAYGVAKQGILIENEERLNWIIRGAVYEPYITIFGNFPTNIDNTLFDISSCSVNASDPLKPKCPMLNADNTPVFPEWLTIMMLCVYLLFANILLLNLLIAIFNYTFQEVQDNTDTIWKFQRYELIKEYHSRPALPPPFILLSHLILFIRGVFLRDLPQRHKNFRQELEQTEEEELLSWEAYMKDNYLASTRQDESQSVEHRIHDTAEKVGAMSELLEREQEMVSATMAKRLARLEEQVSESAKALRWIIDALKSQGCKSKVQPPLMRSKSSDRDDGDSSGQETDDEEAPHMFARQLQYPDSTVRRFPVPEEKVSWEVNFSPYQPPVYNQQDSSESDTSALDKHRNPGGRTGIRGKGALNTLGPNHILHPIFTRWRDAEHKVLEFLAVWEDAEKRWALLGGPAQPDEPLAQVLERILGKKLNEKTKTLLKAGEEVYKGYVDDSRNTDNAWVETSIITLHCDKNTPLMADLNHMVESSLSSHQPLQWREVSSDACRCSYQREALRQIAHHHNTYF |
| Codon-optimized DNA sequence |
| ATGGCCCTGGGCACTAGCGGCGTGAAGATCCACCCTAACGGGAACTCTAACCAGCTGGGGGTGCAGCTGGAGAACGTGAAGCTGACTAGCCTGTTTAAGAAGCTGGACAAGAGGTGTAGCCTCGCCTCTTGGATCAAGGAGAACATTAAGAAGAAGGAGTGCTGCTTCTACGTGGAAGATGGCAGGGAGGGGATCTGCAAGTGCGGGTACCCTAAGGTGCAGCATTGCGATGAGGCCATTAAGCCTGAGGATTACATGGGCGAGCAGTGGGATAAGCACCGGCACGTGAGGGAGACACCCACAGATGCCTTCGGAGACATTAGCTTTGGAGGACTGGGACAGAAGACCGGCAAGTACGTCCGGGTGAGCAGCGACACCTCCTGCGAGAACCTCTACCAGTTGATGACAGAGCAGTGGAAGCTGAGGAGCCCCAACCTGCTGATTAGCGTGACTGGAGGGGCCAAGAACTTTTACATCAAGACCCACCTTAAAGATAAGTTTCGCCGGGGACTGATCAAGGTGGCCCAGACAACAGGGGCCTGGATCTTGACCGGGGGCACCCATGCCGGCGTGATGAAGCACGTGGGGATGGCCGTCAGGGACTACACACTGAGCTCCGGCAGCATGGAGGGCCAGATCGTCGTGATCGGGGTGGCACCCTGGGGCGTGATTCACAACCGGAGCACCCTGATTCACCCCGAGGGGCGGTTCCCCGCCTACTACAGCCTGGATGAGCAGGGCCAGGGGAGGTTGAGCTGCCTTGACATTAACCACACTCACTTCCTCCTGGTGGATGACGGCACACAGGGACACTACGGCGTCGAGATTGAATTGAGGGCCAGGCTGGAGAAGCTGATTTCCAAGCTCAGCTTGGGAAACAGAGAGAGCGGGGTGACCATCCCCGTGGTGTGCGTCGTCCTCGACGGGGGGCCTGGCACCTTGAACACCATCTACAACAGCATGCTGAACCACACTCCCTGCGTGGTCCTTGAGGGCAGCGGACGGCTGGCTGACGTGATTGCCCACGTCGCCAGCGTGCCCGTGTCAAAAGTGACCATGGCCCTCATTAACAGGCTGCTGAAACGCTTCTTCATGCAGGAGTACAAGAACTTTACTGAGTTGCAGATCATCGAATGGACAAAGAAGATCCAGGATATTCTCAGAATGCCACACCTGCTCACAGTCTTTAGGATTGATGAGGATAAGAACTATGACGTGGATGTGGCCATCCTTCAGGCCCTGTTGAAGGCCTCTCGGTCAGACGAGCACGCCGGGCGGCACTGCTGGGAGCGCCAGCTGGAGCTTGCCGTCGCCTGGAACCGCGTGGATATTGCTGAGTCCGAGATTTTTACTGAGGAGAGCCAGTGGACCTCATCCGACCTCCATCCAGCCATGTTTAGTGCTCTCGTGGGCGACAAGCCTGAGTTCGTGAGGCTCCTCCTGGAGAACGGCGTCTGCGTACGGGAGTTTCTTGAGCGGGAGGAGACACTGTGCGAACTGTATAGCCACTTGCCATCCTGCTTCTTCTTGAGGAAGCTGGCCAAGCGCGTCCAGGGGGGGAAGATGCGGAGGGGGCAGGAGCCTCTGCCCGGCAGCCGGAAGGTCTGTCTGAGCCACGTCAGCGAGGAAGTGAGACACCTGCTCGGGAGCTTCACCCAGCCCCTGTACATCGCCTCCAGATACAAGCCCACAAAGGATGACGTGAGGCTCAAAGTCCCTTCCAAGGGCGCCTTGGATCTGCCATGCAGCGGCGAGGAGTGGAGCGCCGACACAGTCTGGGACCCAGGGAGGGACCTCTTCCTGTGGGCCGTGGTCCAGAACAACCGGGAGCTCGCCGAGATTGGCTGGGAGCAGTGTAGAGATTGTATTGCCGCGGCCCTGGCTGCCAGTAAAATTCTCCGGAAGCTGGCCCAGGAGAGCGGCGAGGATGATAGCGAGGAGGCCACCGAGATGTTGGAGCTGGCAAACCATTATGAAAAGCAGGCAATTGGCGTGTTCAGCGAGTGTCATAGTTGGGACGCCCAGAGAGCCCAGAAGCTCTTGATTCGGATCAGCCCTTCCTGGGGCAGGTCCACCTGCCTGTGGCTCGCTCTTGAGGCTCACGACAAGAGCTTCATTGCCCATTCCGGGGTCCAGGCCCTGCTCACCCAGATTTGGTGCGGGGAGCTGAGCGTGGACAACCCTCACTGGAAGGTCTTGCTGTGCATGATCTTCTTCCCCCTGATCTACACAGGCTTCCTGACCTTTAGGAGAGACGAGGATATCCAGCGGCAGGCTGAGAGGACTGAGCAGCAGAAGCTGGCCATGGAGTCCGTGTTCGCCGGGCAGTCCGATGGGAAGATCAAGCGCCATCTTAGGGGCTTTAGCCAGAAGTCTGAGCTGAAGCCACTGAATTGTTCCTCCAGGCTGATGTCCTTCCTGAAGTCCCCTCAGGTGAAGTTTTACTGGAACATCGCCTCCTACTTCGGCTTTCTGTGGCTCTTTGCCGTCGTGCTGATGATTGATTTCCAAACCTCCCCCTCCTGGCGCGAGCTGCTCCTGTACGTCTGGCTGACCAGCCTGGTGTGCGAAGAGATTAGACAGCTGTACCATGACTTTGACGGGAGCGGATTCAGACGGAAGGCCAAGATGTACATTAAGGACCTGTGGAACATTCTTGACGTGCTCTCAATCGTCCTCTTTATCGCCGGACTCATCTGCCGGCTCCAGGCCAGTGACACAGTGTTCTACATCGGCAAAGTGATCCTGTGCATCGATTTCATCATCTTTTGCCTGAGACTGATGGCCATCTTTTCCATTAGCCGGACCCTCGGGCCTAAGATCATTATCGTGCGGCGGATGATGCTGGACCTGTTCTTTTTCATGTTTCTTCTGTCCATTTGGGTCGTCGCATACGGCGTGGCCAAACAGGGCATCCTGATCGAGAACGAGGAGCGGCTCAACTGGATCATCCGGGGCGCCGTGTACGAACCCTATATCACTATCTTCGGCAACTTTCCCACTAACATTGACAACACCCTGTTCGACATCAGCAGCTGCTCCGTGAACGCCTCCGATCCCCTGAAGCCCAAGTGCCCCATGCTGAACGCCGATAACACACCCGTCTTCCCCGAATGGCTGACAATTATGATGCTGTGCGTCTACCTCCTCTTTGCCAACATCCTGCTCCTCAACCTTCTGATCGCTATCTTTAACTACACATTCCAGGAGGTGCAGGACAACACTGACACAATCTGGAAGTTTCAGAGGTACGAGCTGATTAAGGAGTACCACTCCCGGCCCGCCCTGCCCCCCCCCTTCATCCTCCTGTCTCACCTCATTCTGTTCATTAGGGGGGTCTTTCTGAGGGACCTCCCACAGCGGCACAAGAACTTCAGGCAGGAGCTGGAGCAGACTGAGGAAGAGGAGTTGCTCTCCTGGGAGGCCTACATGAAGGATAACTACCTCGCTTCCACAAGACAGGATGAGAGCCAGAGCGTCGAGCACAGGATTCACGACACTGCCGAGAAGGTCGGGGCCATGAGTGAGCTGTTGGAGCGCGAGCAGGAAATGGTGAGCGCTACTATGGCCAAGAGACTGGCCAGGCTGGAGGAGCAGGTGTCCGAGAGCGCCAAGGCCCTGAGGTGGATCATTGATGCCCTGAAGAGCCAGGGGTGTAAGAGCAAGGTGCAGCCTCCACTGATGCGGAGCAAATCTTCCGACCGGGATGATGGAGATAGCTCTGGGCAGGAAACTGATGACGAGGAGGCCCCACACATGTTCGCCCGCCAGTTGCAGTACCCAGACTCCACAGTGCGGAGATTTCCTGTGCCCGAGGAGAAAGTGTCCTGGGAAGTGAACTTTAGCCCTTACCAGCCTCCCGTCTACAACCAGCAGGATTCCAGTGAGTCCGATACATCCGCCCTGGACAAGCATAGGAACCCTGGGGGCCGGACCGGCATCAGGGGAAAGGGCGCCCTGAACACACTGGGGCCAAACCACATTCTCCATCCAATCTTTACTAGATGGAGGGACGCCGAGCACAAGGTGCTGGAGTTCCTGGCCGTCTGGGAGGATGCTGAGAAGCGGTGGGCTCTTCTGGGCGGACCCGCCCAGCCAGACGAGCCCCTGGCCCAGGTCCTGGAGCGGATCTTGGGCAAGAAGTTGAATGAGAAGACCAAGACACTCCTGAAGGCCGGAGAGGAGGTGTACAAGGGGTACGTCGATGATTCACGGAACACTGACAACGCCTGGGTCGAAACAAGCATCATCACCCTGCACTGCGATAAAAACACCCCACTGATGGCTGATCTGAACCACATGGTCGAGAGCAGCCTCAGCTCCCACCAGCCCCTCCAGTGGAGGGAGGTGAGTAGTGATGCCTGTCGGTGCTCCTACCAGAGGGAGGCTCTTAGGCAGATCGCTCACCACCATAACACATACTTC |

**Supplementary Table 1. Sequence of the codon-optimized TRPM2_DR_ gene.**

**Reference**

1 Heymann, J. B. & Belnap, D. M. Bsoft: image processing and molecular modeling for electron microscopy. *J Struct Biol* **157**, 3-18, doi:10.1016/j.jsb.2006.06.006 (2007).
