# Supplementary material for: Visualizing structural transitions of ligand-dependent gating of the TRPM2 channel
Source: Nat Commun. 2019 Aug 20;10:3740. doi: 10.1038/s41467-019-11733-5 (PMC6702222; doi:10.1038/s41467-019-11733-5)
Supplement: Supplementary file 2 — Reporting Summary [file 41467_2019_11733_MOESM2_ESM.pdf]

nature research

Corresponding author(s): Seok-Yong Lee

Last updated by author(s): YYYY-MM-DD

Reporting Summary

Nature Research wishes to improve the reproducibility of the work that we publish. This form provides structure for consistency and transparency in reporting. For further information on Nature Research policies, see Authors & Referees and the Editorial Policy Checklist.

Statistics

For all statistical analyses, confirm that the following items are present in the figure legend, table legend, main text, or Methods section.

n/a	Confirmed	
☐	☒	The exact sample size (*n*) for each experimental group/condition, given as a discrete number and unit of measurement
☐	☒	A statement on whether measurements were taken from distinct samples or whether the same sample was measured repeatedly
☐	☒	The statistical test(s) used AND whether they are one- or two-sided<br>*Only common tests should be described solely by name; describe more complex techniques in the Methods section.*
☐	☒	A description of all covariates tested
☒	☐	A description of any assumptions or corrections, such as tests of normality and adjustment for multiple comparisons
☐	☒	A full description of the statistical parameters including central tendency (e.g. means) or other basic estimates (e.g. regression coefficient) AND variation (e.g. standard deviation) or associated estimates of uncertainty (e.g. confidence intervals)
☐	☒	For null hypothesis testing, the test statistic (e.g. *F*, *t*, *r*) with confidence intervals, effect sizes, degrees of freedom and *P* value noted<br>*Give P values as exact values whenever suitable.*
☒	☐	For Bayesian analysis, information on the choice of priors and Markov chain Monte Carlo settings
☒	☐	For hierarchical and complex designs, identification of the appropriate level for tests and full reporting of outcomes
☒	☐	Estimates of effect sizes (e.g. Cohen's *d*, Pearson's *r*), indicating how they were calculated

Our web collection on statistics for biologists contains articles on many of the points above.

Software and code

Policy information about availability of computer code

Data collection	Clampfit 10.7, Leginon/Appion, EPU, Latitude
Data analysis	Clampfit 10.7, OriginPro 2016, Excel, Leginon/Appion, FindEM, CTFFind4, MotionCor1, MotionCor2, RELION, UCSF Chimera, Coot, Phenix, HOLE, Pymol, CryoSPARC

For manuscripts utilizing custom algorithms or software that are central to the research but not yet described in published literature, software must be made available to editors/reviewers. We strongly encourage code deposition in a community repository (e.g. GitHub). See the Nature Research guidelines for submitting code & software for further information.

Data

Policy information about availability of data

All manuscripts must include a data availability statement. This statement should provide the following information, where applicable:

- Accession codes, unique identifiers, or web links for publicly available datasets
- A list of figures that have associated raw data
- A description of any restrictions on data availability

The sequence of TRPM2DR can be found in the National Center for Biotechnology Information under accession code XP_009303266.1 [https://www.ncbi.nlm.nih.gov/protein/XP_009303266.1?report=genpept]. For the TRPM2DR_Apo-C4, TRPM2DR_Apo-pseudo C4, TRPM2DR_Ca2+, and TRPM2DR_ADPR/Ca2+ structures, the coordinates have been deposited in the Protein Data Bank with the PDB ID 6PKV, 6PKW, 6D73, and 6PKX and the cryo-EM density maps have been deposited in the Electron Microscopy Data Bank with the accession number EMD-20367, EMD-20368, EMD-7822, and EMD-20369. The source data underlying Supplementary Fig. 2 are provided as a Source Data file. Other data are available from the corresponding author upon reasonable request.

Field-specific reporting

Please select the one below that is the best fit for your research. If you are not sure, read the appropriate sections before making your selection.

☒ Life sciences ☐ Behavioural & social sciences ☐ Ecological, evolutionary & environmental sciences

For a reference copy of the document with all sections, see nature.com/documents/nr-reporting-summary-flat.pdf

Life sciences study design

All studies must disclose on these points even when the disclosure is negative.

Sample size	For electrophysiology, sample size represents the number of cells used for recordings. The sizes were chosen based on past experiences and on the numbers required to reach statistic significance.
Data exclusions	For patch-clamp recordings, no data was excluded unless the recording quality was poor due to factors such as large noise and patch instability and leak.
Replication	Electrophysiological experiments were reproduced according to sample size as indicated in each figure.
Randomization	For patch-clamp recordings, samples were grouped based on the genes of interest transfected into the cells.
Blinding	The investigators were not blinded to group allocation.

Reporting for specific materials, systems and methods

We require information from authors about some types of materials, experimental systems and methods used in many studies. Here, indicate whether each material, system or method listed is relevant to your study. If you are not sure if a list item applies to your research, read the appropriate section before selecting a response.

Materials & experimental systems

n/a	Involved in the study	
☒	☐	Antibodies
☐	☒	Eukaryotic cell lines
☒	☐	Palaeontology
☒	☐	Animals and other organisms
☒	☐	Human research participants
☒	☐	Clinical data

Methods

n/a	Involved in the study	
☒	☐	ChIP-seq
☒	☐	Flow cytometry
☒	☐	MRI-based neuroimaging

Eukaryotic cell lines

Policy information about cell lines

Cell line source(s)	HEK293T and HEK293S GnTi- cells were purchased from ATCC.
Authentication	The cell line was authenticated in ATCC and no further authentication was performed.
Mycoplasma contamination	Mycoplasma test was performed by ATCC, and further mycoplasma testing was performed at the start of each new cycle starting from the frozen ATCC cell stock.
Commonly misidentified lines (See ICLAC register)	n/a
